# Supplementary material for: Distinct Classes of Flavonoids and Epigallocatechin Gallate, Polyphenol Affects an Oncogenic Mutant p53 Protein, Cell Growth and Invasion in a TNBC Breast Cancer Cell Line
Source: Cells. 2021 Apr 2;10(4):797. doi: 10.3390/cells10040797 (PMC8067228; doi:10.3390/cells10040797)
Supplement: Supplementary file 1 [file cells-10-00797-s001.pdf]

Supplementary Figure 1 (related to Figure 2)

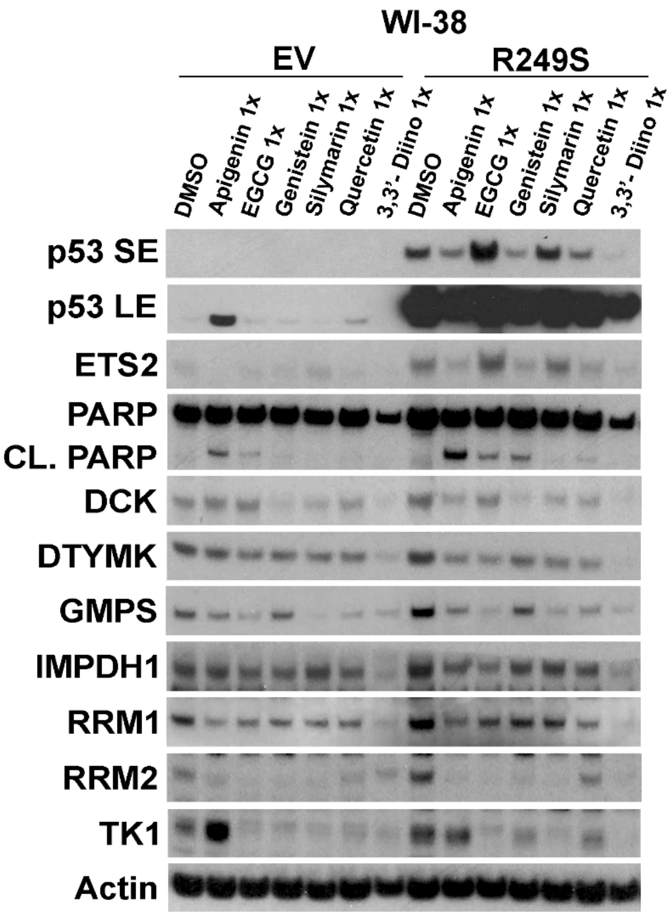

Supplementary Figure 1. Effect of plant derived compounds in the presence of wildtype or mutant p53**.** WI38 cells were treated with 1x IC50 (concentration as used for mutant p53 cell line, BT-549) concentration for 24 hours. Cells were lysed and processed for western blot analysis. R249S mutant was stably expressed using a lentiviral system. SE: Shorter exposure, LE: Longer exposure. CL. PARP: Cleaved PARP.

Supplementary Figure 2 (related to Figure 4)

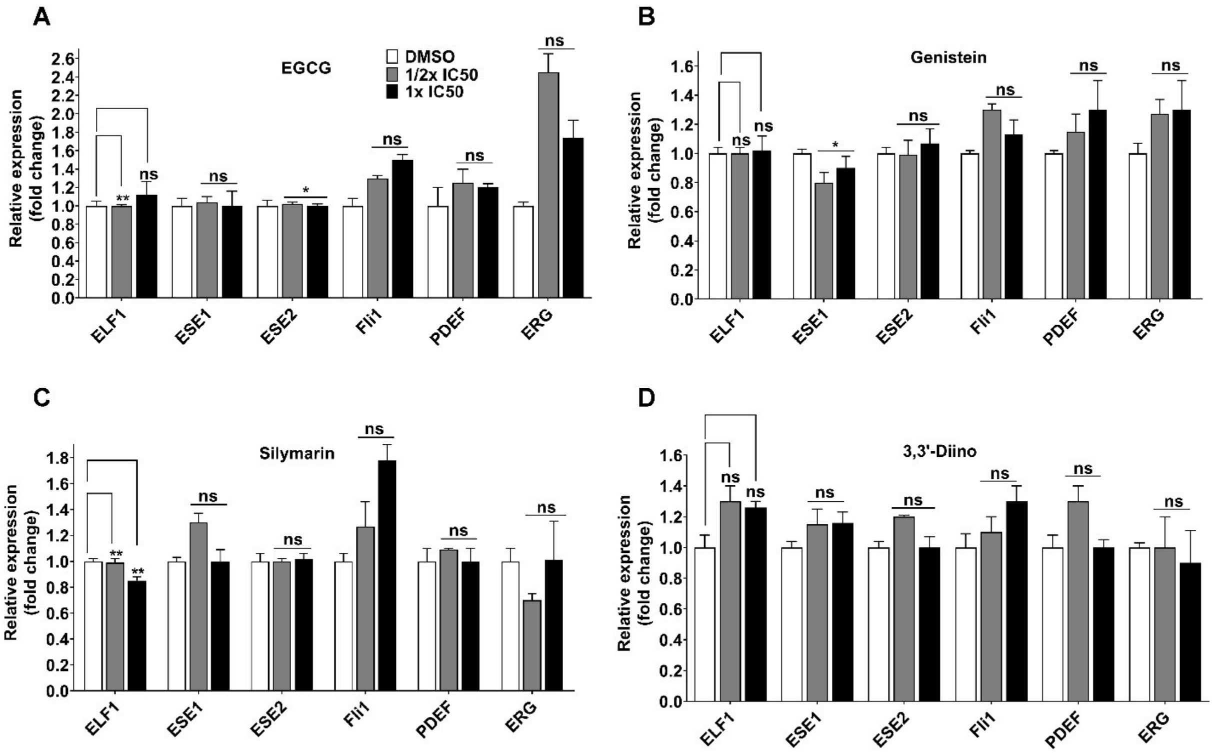

Supplementary Figure 2. Modulation of other ETS members in response to plant derived compounds. BT-549 cells were treated with 1/2x and 1x IC50 values for 24 hours. RNA was extracted to prepare cDNA. qRT-PCR was performed for several other ETS family members. After the treatment with EGCG **(A)**, Genistein **(B)**, Silymarin **(C)**, and 3,3′-Diino **(D)** modulation of other ETS members can be seen. Errors bars represent mean ±SD derived from three independent replicates. *, **, and *** denote $p < 0.05$, $p < 0.01$, and $p < 0.001$, respectively. "NS" means not significant.

Supplementary Table 1: IC50 values (µM) of natural compounds derived from three independent replicates (n=3).

IC50 (µM)	BT-549	HCC38	MDA-MB-231
Apigenin	26 (±3.20)	33 (±5.20)	45 (±6.10)
EGCG	163 (±14.00)	150 (±19.3)	>500 (NA)
Genistein	25 (±2.89)	88 (±9.91)	>100 (NA)
Silymarin	88 (±8.80)	>100 (NA)	>100 (NA)
Quercetin	27 (±3.97)	29 (±2.23)	82 (±5.53)
3,3′-Diino	29 (±1.99)	>100 (NA)	45 (±4.33)

*Values in parentheses represent standard deviation calculated from three independent experiments.

Supplementary Table 2: Real-Time qRT-PCR primer sequences

Gene	Primer sequence
ADSS rt f	TAC TGT TGG AGG TGT TTG TA
ADSS rt r	CCC CTT GTT TGT AAT AAT TC
DCK rt f	GCCAGATGGTGCAATGTTC
DCK rt r	GCATCTTTGAGCTTGCCATT
DCTD rt f	AAC ATG AGT GAA GTT TCC TG
DCTD rt r	TTC TCT GTG CTG ATA AGA AG
DHFR rt f	TCC CAG ACA GAA CCT ACT AT
DHFR rt r	CGA TTC TTC CAG TCT ACG
DTYMK rt f	CTA CTT GCA AAA GAA AAG TG
DTYMK rt r	GCT GTT TAC ACC AAT CTA GG
GMPS rt f	GGTGGAGTAGACTCAACAGT
GMPS rt r	CATTGTAGAAAGAATGAGCA
IMPDH1 rt f	CTAGATTGGACCTCGCTACA
IMPDH1 rt r	GATCCATCTGGACACCAAC
IMPDH2 rt f	CTCACCTACAATGACTTTCTC
IMPDH2 rt r	GGGTCTTAAGAGTGATTTTC
RRM1 rt f	AGA AGA TTG CAA AGT ATG GT
RRM1 rt r	GTA AGG TTC AAT GGA CTC AT
RRM2 rt f	TAG AGG TGG TTC CTA CAA GT
RRM2 rt r	GGG TGA CTG AAG TAT GAA CT
RRM2B rt f	CTGGGTGCTGTCGTAGTT
RRM2B rt r	TGGCTCTTCATTTGACTTTA
TK1 rt f	CAATGAGCTGCATTAACCT
TK1 rt r	GTGTCTTTGGCATACTTGAT
TYMS rt f	CTA CAG CCT GAG AGA TGA AT
TYMS rt r	GAA GAC AGC TCT TTA GCA TT
P53 rt f	TGCTTTCCACGACGGTGACA
P53 rt r	ATCTGACTGCGGCTCCTCCA

ETS1 rt f	TGAGACACGACCTAAGTTGAAGAGTT
ETS1 rt r	TTCCAAAGACAGCCAAGTCACATT
ETS2 rt f	GGAGCCATTCATTCGGAGAAAA
ETS2 rt r	CCTGTCGGACTTTGAAACCAACTT
ETV1 rt f	GCCGTTCACTCCGCTATTAC
ETV1 rt r	CTGTGTCCTCCTCGTTGATG
ETV4 rt f	AGGAGACGTGGCTCGCTGA
ETV4 rt r	GGGGCTGTGGAAAGCTAGGTT
ETV5 rt f	ACCAATGGGAATCAAGCAGGA
ETV5 rt r	GGATGACTGGCAGTTAGGCA
HPRT rt f	CAGACTTTGCTTTCCTTGGTCAG
HPRT rt r	CACTTCGTGGGGTCCTTTTCAC

Supplementary Table 3. Definitions of genes

Gene symbol	Definition
ADSS	Adenylosuccinate synthase
DCK	Deoxycytidine kinase
DHFR	Dihydrofolate reductase
DTYMK	Deoxythymidylate kinase
GMPS	Guanosine monophosphate synthase
IMPDH1	Inosine monophosphate dehydrogenase 1
IMPDH2	Inosine monophosphate dehydrogenase 2
RRM1	Ribonucleotide reductase M1
RRM2	Ribonucleotide reductase M2
RRM2b	Ribonucleotide reductase M2 B
TK1	Thymidine kinase 1
TYMS	Thymidylate synthetase
ETS1	v-ets avian erythroblastosis virus E26 oncogene homolog 1
ETS2	v-ets avian erythroblastosis virus E26 oncogene homolog 2
ELF1	E74-like factor 1 (ets domain transcription factor)
ESE1	Epithelium-specific Ets transcription factor 1
ESE2	Epithelium-specific Ets transcription factor 2
ETV1	ets variant 1
ETV4	ets variant 4
ETV5	ets variant 5
Fli1	Fli-1 proto-oncogene, ETS transcription factor
PDEF	Prostate-derived Ets factor
ERG	v-ets avian erythroblastosis virus E26 oncogene homolog
